# Quinone-dependent D-lactate dehydrogenase Dld (Cg1027) is essential for growth of *Corynebacterium glutamicum *on D-lactate

**DOI:** 10.1186/1471-2180-10-321

**Published:** 2010-12-15

**Authors:** Osamu Kato, Jung-Won Youn, K Corinna Stansen, Daisuke Matsui, Tadao Oikawa, Volker F Wendisch

**Affiliations:** 1 Department of Biotechnology, Faculty of Engineering, Kansai University, 3-3-35 Yamate-Cho, Suita, Osaka-Fu 564-8680, Japan; 2 Chair of Genetics of Prokaryotes, Department of Biology & CeBiTec, Bielefeld University, Bielefeld, Germany; 3 Institute of Molecular Microbiology and Biotechnology, Westfalian Wilhelms University Muenster, Muenster, Germany

## Abstract

**Background:**

*Corynebacterium glutamicum *is able to grow with lactate as sole or combined carbon and energy source. Quinone-dependent L-lactate dehydrogenase LldD is known to be essential for utilization of L-lactate by *C. glutamicum*. D-lactate also serves as sole carbon source for *C. glutamicum *ATCC 13032.

**Results:**

Here, the gene cg1027 was shown to encode the quinone-dependent D-lactate dehydrogenase (Dld) by enzymatic analysis of the protein purified from recombinant *E. coli*. The absorption spectrum of purified Dld indicated the presence of FAD as bound cofactor. Inactivation of *dld *resulted in the loss of the ability to grow with D-lactate, which could be restored by plasmid-borne expression of *dld*. Heterologous expression of *dld *from *C. glutamicum *ATCC 13032 in *C. efficiens *enabled this species to grow with D-lactate as sole carbon source. Homologs of *dld *of *C. glutamicum *ATCC 13032 are not encoded in the sequenced genomes of other corynebacteria and mycobacteria. However, the *dld *locus of *C. glutamicum *ATCC 13032 shares 2367 bp of 2372 bp identical nucleotides with the *dld *locus of *Propionibacterium freudenreichii *subsp. *shermanii*, a bacterium used in Swiss-type cheese making. Both loci are flanked by insertion sequences of the same family suggesting a possible event of horizontal gene transfer.

**Conclusions:**

Cg1067 encodes quinone-dependent D-lactate dehydrogenase Dld of *Corynebacterium glutamicum*. Dld is essential for growth with D-lactate as sole carbon source. The genomic region of *dld *likely has been acquired by horizontal gene transfer.

## Background

Lactate is a major product of anaerobic metabolism. D-, L, and DL-lactic acid can be utilized by anaerobic and aerobic microorganisms as a carbon and energy source. Propionibacteria preferentially ferment L-lactate to propionate, acetate and carbon dioxide [1], *Eubacterium hallii *ferments both lactate isomers to butyrate in the human colon [2], while D-lactate is fermented to acetate by sulfate-reducing bacteria such as *Desulfovibrio vulgaris *[3], or to butyrate by e.g. *Clostridium indolis*-related strains isolated from human feces [2]. D-lactic acidosis in humans, which can lead to neurotoxicity and cardiac arythmia, is associated with an imbalance of production and degradation of D-lactate by the colonic microbiome [4]. D-lactate oxidizing enzymes have been described in eukaryotes and bacteria [5-8]. In *Escherichia coli *two membrane associated oxidizing lactate dehydrogenases are known. LldD is specific for L-lactate and is not able to oxidize D-lactate as substrate, meanwhile the second Lactate dehydrogenase Dld shows high affinity to D-lactate but also low affinity activity with L-lactate. Both enzymes convert lactate to pyruvate by removing electrons from lactate to enter the electron transport chain.

Peptidoglycan precursors may contain D-lactate as the C-terminal D-alanine residue of the muramyl pentapeptide is replaced by D-lactate, known as a pentadepsipeptide. This pentadepsipeptide is the cause of the acquired resistance of pathogenic enterococci to vancomycin and of the natural resistance of several lactobacilli to this glycopeptide antibiotic [9]. In *L. plantarum*, D-lactate for peptidoglycan precursor synthesis can be provided by the NAD-dependent fermentative D-lactate dehydrogenase or by a lactate racemase, which is encoded by an L-lactate-inducible operon, or by addition of D-lactate to the medium [10]. In *E. coli*, D-lactate can be generated during cell wall recycling and during growth on N-acetylmuramic acid as the etherase MurQ cleaves N-acetylmuramic acid 6-phosphate to yield N-acetylglucosamin 6-phoshate and D-lactate [11,12].

The uptake of lactate can be mediated by different kinds of transporters. The uptake systems LldP and GlcA, members of the lactate permease LctP family, are responsible for the uptake of DL-lactate and glycolate in *E. coli *[13]. In *Rhizobium leguminosarum *uptake of lactate and pyruvate, respectively, is mediated by MctP [14]. MctP belongs to the family of solute:sodium symporter (SSS).

*C. glutamicum*, a gram-positive facultative anaerobic bacterium is used for the biotechnological amino acid production in the million-ton-scale [15]. This bacterium can use a variety of carbon sources for growth, e.g. sugars like glucose, fructose and sucrose, organic acids like citrate, gluconate, pyruvate, acetate and propionate, but also ethanol, glutamate, vanillate or 4-hydroxybenzoate [16-23]. With two exceptions, namely glutamate and ethanol, carbon sources are utilized simultaneously by *C. glutamicum*. L-lactate and D-lactate are also known as sole or combined carbon sources of *C. glutamicum *[24]. MctC, a member of the solute:sodium symporter family recently identified and characterized, catalyzes the uptake of the monocarboxylates acetate, pyruvate and propionate, but there is no indication of a MctC dependent uptake of lactate in *C. glutamicum *[25].

Utilization of L-lactate by *C. glutamicum *has been studied to some detail and requires quinone-dependent L-lactate dehydrogenase LldD (EC 1.1.2.3) which is encoded by the cg3226-*lldD *operon [24]. Although cg3226 encodes a putative lactate permease, it is not required for growth in L-lactate minimal medium [20]. Expression of the cg3226-*lldD *operon is maximal when L-lactate is present in the medium. The cg3226-*lldD *operon is repressed by the FadR-type transcriptional regulator LldR in the absence of its effector L-lactate [20]. LldR is also known to repress the fructose utilization operon *fruR-fruK-ptsF *[26] and the gene for the fermentative NAD-dependent L-lactate dehydrogenase *ldhA *[27].

Relatively little is known about utilization of D-lactate by *C. glutamicum*. Only the production of D-lactate has been demonstrated with *C. glutamicum *R by heterologous expression of fermentative D-lactate dehydrogenase (D-LDH)-encoding genes from *Lactobacillus delbrueckii *and *Escherichia coli *[28]. The analysis of D-lactate utilization by *C. glutamicum *is important with respect to biotechnological D-lactate production, to further understanding of its physiology and with respect to the so-called flexible feedstock concept. Therefore, this study aimed to identify and characterize gene(s) and enzyme(s) for D-lactate utilization by this bacterium.

## Methods

### Bacterial strains, plasmids, oligonucleotides, and culture conditions

Used Bacterial strains, plasmids and oligonucleotides are listed in Table 1. *E. coli *and *Corynebacterium *strains were grown on Luria-Bertani (LB) medium as complex medium [29]. For growth experiments with *C. glutamicum *and *C. efficiens*, in the first preculture, 50 ml LB medium was inoculated from a fresh LB agar plate and incubated at 30°C and 120 rpm. After washing the cells in 0.9% NaCl (w/v), the second preculture and the main culture were inoculated to an optical density at 600 nm (OD_600_) of 0.5 to 1.0 in 50 ml CgXII minimal medium [30], which contained 0.03 g/l protocatechuic acid. As carbon and energy sources, 100 mM glucose, 100 mM sodium L-lactate, 100 mM sodium D-lactate or 50 mM sodium L-lactate and 50 mM sodium D-lactate were used. Precultures and main cultures were incubated at 30°C and 120 rpm on a rotary shaker in 500 ml-baffled shake flasks. When appropriate, 1 mM isopropyl-β-D-thiogalactopyranosid (IPTG), kanamycin (25 μg/ml) or spectinomycin (100 μg/ml) was added to the media. Growth of *C. glutamicum *and *C. efficiens *was followed by measuring the OD_600_. For all cloning purposes, *Escherichia coli *DH5α was used as host.

**Table 1:** List of bacterial strains, plasmids and oligonucleotides

strain, plasmid or oligonucleotide	relevant characteristics or sequence	source or reference
***E. coli *strains**		
DH5α	F^- ^*thi*-1 *endA1 hsdR17*(r^- ^m^-^) *supE44 *Δ*lacU169 *(ϕ80*lacZ*ΔM15) *recA1 gyrA96 relA1*	[32]
***Corynebacterium *strains**		
*C. glutamicum* ATCC 130302		ATCC [61]
::*dld*	*dld *inactivation mutant of ATCC 13032	This work
*C. efficiens* DSM44547		DSM
		
**Plasmids**		
pEKEx3	Spec^R^; P_tac_, *lacI*^q^	[24]
pEKEx3-*dld*	pEKEx3 containing *dld *from *C. glutamicum *and an artificial ribosome binding site	this work
pVWEx1	Kan^R^; P_tac_, *lacI*^q^	[34]
pVWEx1-*dld*	pEKEx3 containing *dld *from *C. glutamicum *and an artificial ribosome binding site	This work
pK18*mob*	Kan^R^; integration vector for *C. glutamicum*	[62]
pK18*mob*-*dld*	pK18*mob *carrying an internal fragment of *dld*	This work
		
**Oligonucleotides**	**sequence**	**underlined nucleotide** **(position in NC003450)**
rbs-ndld	GTCGAC**AAGGAGATATAGAT**ATGACGCAACCAGGACAGAC SalI	955683
cdld	GGAGCTCTTAGGCCCAGTCCTTGTGC SacI	957398
Ex-*dld*-fw	GC*CCTGCAGG***AAGGAGATATAGAT**ATGACGCAACCAGGACAGAC SbfI	955683
Ex-*dld*-bw	GC*GGTACC*TTAGGCCCAGTCCTTGTGC KpnI	957398
*dld1*	AATCATATGAGACGCAACCAGGACAGACCACC NdeI	955683
*dld2*	AATGGATCCGGCCCAGTCCTTGTGCGGCGACGTGC BamHI	957398
Cg-*dld*-SalI-N498	AAGTCGACAGCCAGATTCCAGATTCGCAAGGGT SalI	956907
Cg-*dld*-C1716-SalI	GGTCGACTTAGGCCCAGTCCTTGTGC SalI	955683

### Determination of glucose and D/L-lactate concentrations

During cultivation, samples (1 ml) were collected to determine biomass, glucose and D/L-lactate concentrations. After determinations of the OD_600 _and centrifugation of the sample (13,000 g, 5 min) aliquots of the supernatant were used to determine concentrations of glucose and D/L-lactate by reverse-phase high-pressure liquid chromatography (HPLC) as described by Engels *et al. *2008. To discriminate between the D- and L- isomers of lactate enzymatic determinations were performed as described by the manufacturer (R-Biopharm, Darmstadt, Germany).

### D-lactate dehydrogenase assay

For determination of enzyme activities, exponentially growing cells were harvested by centrifugation (4,500 g, 5 min, 4°C) and washed twice with 50 mM ice-cold KH_2_PO_4_, pH 7.0. Cell pellets were resuspended in 1 ml of 50 mM KH_2_PO_4_, pH 7.0, directly or after storage at -70°C. After disruption by ultrasonic treatment at 4°C (UP 200S; Dr. Hielscher GmbH, Teltow, Germany) at an amplitude of 55% and a duty cycle of 0.5 for 6 min and centrifugation at 4°C for 60 min at 13,000 g, enzyme activity was determined immediately in the cell-free supernatant. D-Lactate dehydrogenase activity was determined by a modified assay according to [31]. Reaction mixtures of 1 ml contained 100 mM KH_2_PO_4 _(pH 7.5), 50 μM 2,6-dichloroindophenol (DCPIP) and 20 μl crude extract. The reaction was started by addition of 10 mM D-lactate and quinone-dependent D-lactate dehydrogenase was assayed spectrophotometrically at 30°C by determining the decrease in absorbance of DCPIP (ε_600 _= 20 mM^-1 ^cm^-1^).

### Construction of plasmids and strains

The oligonucleotides listed in Table 1 were obtained from Operon (Cologne, Germany). Standard methods such as PCR, restriction, and ligation were carried out as described previously [29]. Plasmids were constructed in *Escherichia coli *DH5α from PCR-generated fragments (KOD, Novagen) and isolated with the QIAprep spin miniprep kit (QIAGEN, Hilden, Germany). *E. coli *was transformed by the RbCl_2 _method [32], while *C. glutamicum *was transformed via electroporation [33]. All cloned DNA fragments were shown to be correct by sequencing (BigDye Terminator v3.1 Cycle Sequencing Kit and ABI Prism Capillary Sequencer Model 3730, Applied Biosystems, Forster-City, USA).

### Disruption of *dld*

To construct a *C. glutamicum dld *inactivation mutant, an internal 1224-bp fragment of *dld *was amplified by using primer pair Cg-*dld*-SalI-N498 and Cg-*dld*-C1716-SalI which was subsequently cloned into pT7-blue T-vector (Novagen). The SalI restricted PCR fragment was ligated into the SalI site of pK18*mob*. Gene inactivation with pk18*mob*N498*dld *was carried out as described previously [24]. The correct genotype of the insertion mutant was verified by PCR analysis and determination of enzyme activity.

### Homologous overexpression of *dld*

For homologous overexpression *dld *was amplified from genomic DNA of *C. glutamicum *WT by using primers rbs-ndld and cdld and was cloned into the expression vector pEKEx3 [24]. The amplified PCR fragment was ligated to a SmaI bluntend restriction site of pEKEx3. The constructed vector pEKEx3-*dld *allows the IPTG-inducible expression of *dld *in *C. glutamicum*. Because *C. efficiens *could not be transformed with pEKEx3-*dld*, *dld *was amplified using the primer Ex-*dld*-fw and Ex-*dld*-bw. The PCR fragment was cloned into the expression vector pVWEx1 [34] via SbfI and KpnI restriction sites. The vector pVWEx1-*dld *was transformed into *C. effiens *by electroporation and allowed IPTG-inducible expression of *dld *in this species.

### Expression of *dld *from *C. glutamicum *ATCC 13032 in *Escherichia coli *BL21 (DE3)

Based on the 5'- and 3'- sequences of *dld *(accession no. YP_225194) in the genomic DNA of *Corynebacterium glutamicum *ATCC 13032, the oligonucleotides *dld1 *and *dld2 *were designed, and *dld *was amplified by PCR from the genomic DNA of *C. glutamicum *ATCC 13032 (1 ng) with *dld1*and *dld2 *(0.2 pmol). The thermal profiles for PCR involved the denaturation (94°C for 5 min), 5 cycles of annealing1 (98°C for 10 sec, 58°C for 30 sec, and 72°C for 90 sec) and subsequently 20 cycles of annealing 2 (98°C for 10 sec, 60°C for 30 sec, 72°C for 90 sec), and the extension (72°C for 7 min). A PCR amplification was carried out with a Blend Taq polymerase in a Gene Amp PCR system 9700 (PE Applied Biosystems, Piscataway, NJ, USA). The resulting 1,020-bp fragment with NdeI and BamHI restriction sites was sequenced with a DNA sequencing system, SQ5500 (Hitachi, Tokyo,). The obtained *dld *was ligated into an NdeI and BamHI-digested pT7 Blue-2 T-vector (50 ng/μl) and transformed into *E. coli *NovaBlue. After cultivation in an LB medium containing ampicillin, the plasmid was extracted with the alkaline mini-prep method and precipitated with polyethylene glycol 6,000. The purified DNA obtained was digested with NdeI and BamHI, and ligated into an NdeI and BamHI-restricted pET14b vector to form pET14b-*dld*. pET14b-*dld *was transformed into *E. coli *BL21 (DE3).

### Expression of *dld *in *E. coli *BL21 (DE3) and protein purification

After the *E. coli *BL21 (DE3) cells harboring pET14b-*dld *were selected on an LB agar medium containing ampicillin (100 μg/ml), two clones were inoculated into a LB medium (5 ml) containing ampicillin (100 μg/ml) and cultivated at 30°C until the turbidity at 600 nm reached to 0.4-0.8. The culture was inoculated into the same medium (1 l) and cultivated at 30°C for 14 h. The cells were collected by centrifugation (7,100 × *g*, 10 min), suspended in 0.85% (w/v) NaCl, and centrifuged again. The cells were resuspended in a 20 mM sodium phosphate buffer (pH 8) containing 300 mM NaCl (Buffer A) and stored at -20°C. The cells were disrupted by ultrasonication (model UD-201, Tomy Seiko CO., Tokyo). The disruption conditions used were as follows: output 6; duty cycle 30; and operation time 5 min × 10 times. The suspension was ultracentrifuged (161,000 × *g*, 1 h), and the supernatant was dialyzed against Buffer A. The pellet obtained was suspended in Buffer A plus 0.5% Triton X-100 (Buffer B) at room temperature. After 1 h, the suspension was ultracentrifuged (161,000 × *g*, 1 h), and the supernatant obtained was stored at 4°C. The cell-free extract solubilized (about 120 mg) was applied to a column of TALON metal affinity resin (TaKaRa Bio, Inc. (Shiga, Japan)**; **10 × 15 cm). The column was equilibrated with Buffer B at a flow rate of 0.5 ml/min, and washed successively with Buffer B (90 ml), Buffer B plus 10 mM Imidazole (16 ml), Buffer B plus 20 mM Imidazole (16 ml), and Buffer B plus 50 m M Imidazole (4 ml). The adsorbed protein was eluted with Buffer B plus 250 mM imidazole (20 ml). The elution was collected with a Bio-collector (ATTO, Tokyo. Japan, 2 ml/tube), and the protein concentration was measured with a RC DC Protein assay kit (Bio-Rad Laboratories, Inc., Hercules, CA, USA). The fractions containing the D-lactate dehydrogenase were dialyzed against two 1-l portions of Buffer A for 4 and 12 h, and stored at 4°C.

### Comparative transcriptome analysis using DNA microarrays

Generation of *C. glutamicum *whole-genome DNA microarrays, total RNA preparation, synthesis of fluorescently labelled cDNA, microarray hybridization, washing, and statistical data analysis were performed as described previously [35-38]. Genes exhibiting mRNA levels that were significantly changed (P ≤ 0.05 in Student's *t *test) by at least a factor of 2.0 were determined in three DNA microarray experiments performed with RNA isolated from three independent cultures. The processed and normalized data have been deposited in the NCBI's Gene Expression Omnibus and are accessible under the accession number GSE25704.

## Results

### Cg1027 encodes D-lactate dehydrogenase

The *C. glutamicum *ATCC 13032 gene cg1027 was annotated to code for D-lactate dehydrogenase [39] as the deduced protein shows similarities to FAD/FMN-containing dehydrogenases encoded by the cluster of orthologous genes COG0277. The deduced protein contains the conserved domain PRK11183, and the domain (aa 279-570) was similar to membrane-binding D-lactate dehydrogenases belonging to the protein family pfam09330.

In order to determine whether the gene product of cg1027 is indeed active as D-lactate dehydrogenase, the gene was cloned into pET14b, and the hexahistidine-tagged protein was purified from *E. coli *BL21 (DE3) harboring pET14b-*dld*. Quinone-dependent D-lactate dehydrogenase activity was detected by using 2,6-dichloroindophenol as an electron acceptor. The optimum assay conditions were observed in a 100 mM potassium phosphate buffer at a pH of 7.0 and a temperature of 45°C. Subsequently, Dld activity was assayed at 30°C, the optimal temperature for growth of *C. glutamicum*. The enzyme showed Michaelis-Menten kinetics with D-lactate as the substrate and it was determined that 0.61 mM of D-lactate resulted in half maximal enzyme activity. The observed *V_max _*was 73.5 μmol mg^-1. ^min^-1^. When D-lactate was replaced as substrate, the enzyme did not significantly act with D-malate, L-malate, D-tartrate and L-tartrate, but some oxidation of L-lactate and DL-2-hydroxybutyrate was observed ( < 5% of the activity observed for D-lactate). The comparison of the absorption spectra of the purified protein from *C. glutamicum *with those of the NAD-dependent D-lactate dehydrogenase from *Leuconostoc mesenteroides *revealed that the absorption maxima at 375 nm and 445 nm were observed only for the protein from *C. glutamicum*. These spectral features agree well with those for FAD. Moreover, the primary structure of the D-lactate dehydrogenase from *C. glutamicum *contains a domain (aa 50-187) similar to the FAD binding domain 4 found in the members of the protein family pfam01565. These results suggest that D-lactate dehydrogenase from *C. glutamicum *contains FAD as a bound cofactor. Taken together, it is concluded that cg1027 encodes quinone-dependent D-lactate dehydrogenase (EC 1.1.2.4) from *C. glutamicum *and, thus, was named *dld*.

### Dld is required for utilization of D-lactate

**Figure 1:**
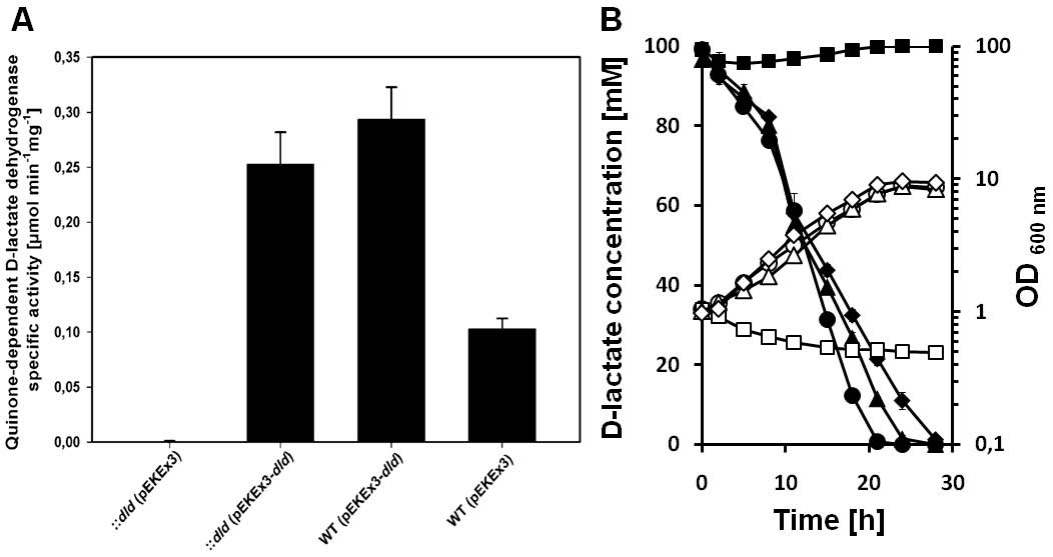
**Specific activities of the quinone-dependent D-lactate dehydrogenase Dld (A) and growth (B) of various *C. glutamicum *strains**. Specific Dld activities (A) were determined after growth in LB complex medium containing 1 mM IPTG. The values represent means and standard deviations of at least three independent cultivations. Growth (B) of *C. glutamicum *WT(pEKEx3) (diamonds), WT(pEKEx3-*dld*) (circles), ::*dld*(pEKEx3) (squares), and ::*dld*(pEKEx3-dld) (triangles) in CgXII mineral medium containing 100 mM D-lactate and 1 mM IPTG was monitored as OD_600nm _(open symbols). The concentration of D-lactate in the supernatant was measured by HPLC (closed symbols). Averages and experimental errors from at least three independent growth experiments are shown.

In order to determine the role of quinone-dependent D-lactate dehydrogenase Dld for growth of *C. glutamicum *on D-lactate and racemic DL-lactate, a defined *dld *disruption mutant and *dld *overexpression plasmids for complementation were constructed. The constructed strains were assayed for Dld activity in crude extracts obtained after growth in LB medium containing kanamycin and IPTG when appropriate. Crude extracts of *C. glutamicum *WT and WT(pEKEx3) contained about 0.10 U mg^-1 ^Dld activity (Figure 1), while no Dld activity was detectable in *C. glutamicum *::*dld *(pEKEx3). Overexpression of *dld *resulted in about three fold higher Dld activity in WT(pEKEx3-*dld*) than in the empty vector control. Growth experiments with *C. glutamicum *strains WT(pEKEx3), WT(pEKEx3-*dld*), ::*dld*(pEKEx3), and ::*dld*(pEKEx3-*dld*) in CgXII mineral medium containing 100 mM D-lactate and 1 mM IPTG revealed that *dld *is required for growth of *C. glutamicum *on D-lactate as sole carbon and energy source as only strains with intact *dld *either on the chromosome or on plasmid could grow (Figure 1).

In media containing 100 mM racemic DL-lactate as carbon and energy source, *C. glutamicum*::*dld*(pEKEx3) formed about half as much biomass as strains WT(pEKEx3), WT(pEKEx3-*dld*), and ::*dld*(pEKEx3-*dld*) indicating that only L-lactate is utilized in the absence of Dld while strains possessing Dld utilized both L- and D-lactate for growth (data not shown).

### Dld activities under various growth conditions

**Figure 2:**
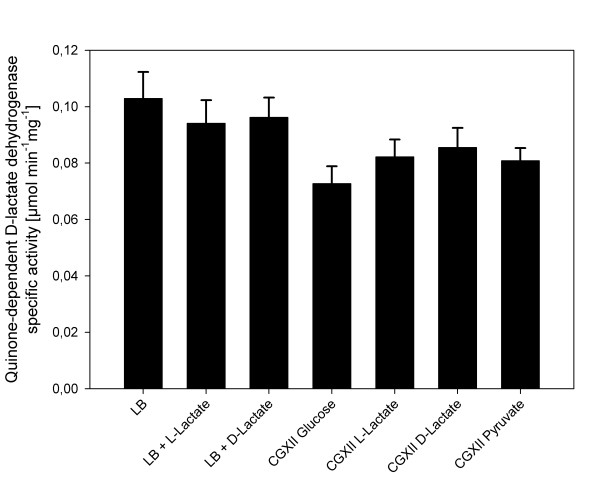
**Specific activities of the quinone-dependent D-lactate dehydrogenase Dld in crude extracts of *C. glutamicum *WT grown in different media**. The values represent means and standard deviations of at least three independent cultivations in LB complex medium without or with 100 mM L-lactate or 100 mM D-lactate or in CgXII mineral medium containing either 100 mM glucose, 100 mM L-lactate, 100 mM D-lactate or 100 mM pyruvate as carbon source.

The specific quinone-dependent D-lactate dehydrogenase activity was determined in crude extracts of *C. glutamicum *ATCC 13032 grown under different conditions. Neither the addition of L-lactate nor of D-lactate to complex medium affected the specific activity of Dld (Figure 2). Dld activities were also similar after growth in CgXII minimal medium with various carbon sources (Figure 2). Thus, the comparable Dld activities in *C. glutamicum *cells grown in different media suggested that *dld *is expressed constitutively.

### DNA microarray analysis of D-lactate specific gene expression changes

Comparative transcriptome analysis was performed for *C. glutamicum *cells grown in LB with/without added D-lactate as well as in CgXII minimal medium with DL-lactate or L-lactate as sole carbon sources. These carbon source combinations were chosen to avoid secondary effects in comparisons with non-gluconeogenic carbon sources such as glucose and because L-lactate specific gene expression patterns were known [24]. Neither the addition of D-lactate to LB nor the presence of D-lactate in minimal medium affected *dld *expression. However, upon addition of D-lactate to LB medium eight genes showed altered expression levels as compared to the absence of D-lactate. Of these, five genes showed higher and three genes lower RNA levels in the presence of D-lactate. Growth in DL-lactate minimal medium was characterized by lower expression of fourteen genes as compared to growth in L-lactate. As most of these genes encoded ATPase subunits or ribosomal proteins this expression pattern likely reflects the lower growth rate in DL-lactate than in L-lactate minimal medium.

### Heterologous expression of *dld *from *C. glutamicum *ATCC 13032 in *C. Efficiens*

**Figure 3:**
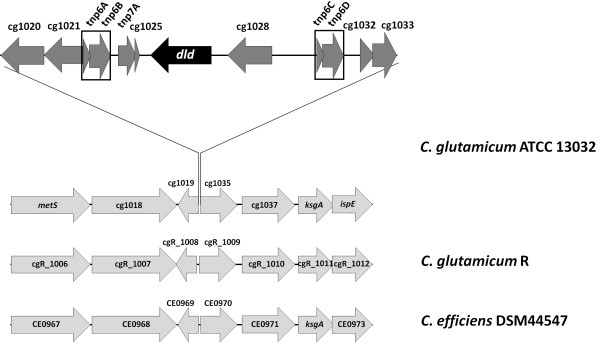
**Comparison of the genomic context of *dld *in *C. glutamicum *ATCC13032 with the closely related *C. glutamicum *R and *C. efficiens *DSM44547**. An insertion of twelve genes (including *dld*) is present only in the genome of *C. glutamicum *ATCC 13032. The regions flanking this genomic island are homologous to those in *C. glutamicum *R and *C. efficiens*. Direct repeats are located close to *dld *and are marked with boxes. The data were obtained from the open source bioinformatics tools CoryneRegNet [63] and PRODORIC Database [64].

**Figure 4:**
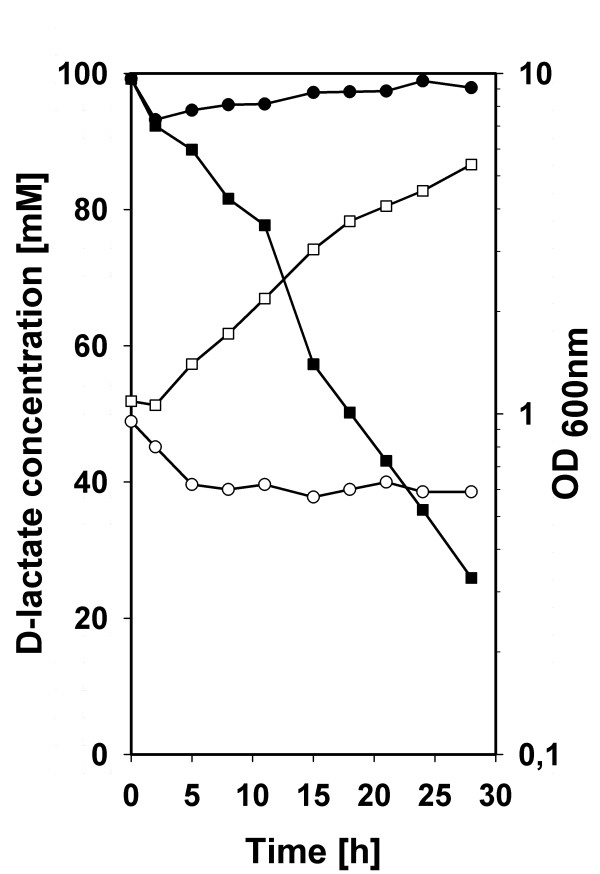
**Growth of *C. efficiens *DSM44547 carrying either the empty vector pVWEx1 (squares) or the vector pVWEx1-*dld *(circles) in CgXII mineral medium containing 100 mM D-lactate and 1 mM IPTG**. A representative growth curve is shown. The growth was monitored as OD_600nm _(closed symbols); the concentration of D-lactate in the supernatant was measured by HPLC (open symbols).

Comparison of the genome of *C. glutamicum *ATCC 13032 with the genomes of closely related species revealed that *C. glutamicum *R, *C. efficiens*, *C. jeikeium *and *C. urealytikum *do not possess a protein homologous to Dld (Figure 3). *C. efficiens *has been described to be unable to assimilate D-lactate [40]. To test whether the absence of a gene homologous to *dld *resulted in the inability of *C. efficiens *to grow in D-lactate minimal medium, *C. efficiens *DSM44547 was transformed either with the empty vector pVWEx1 or the *dld *expression vector pVWEx1-*dld *and growth of *C. efficiens *strains DSM44547, DSM44547(pVWEx1) and DSM44547(pVWEx1-*dld*) was analysed in CgXII mineral medium containing 100 mM D-lactate and 1 mM IPTG. As expected [40], *C. efficiens *strains DSM44547 and DSM44547(pVWEx1) could not grow with D-lactate as sole carbon source (data not shown and Figure 4), while *C. efficiens *ATCC DSM44547(pVWEx1-*dld*) utilized D-lactate for biomass formation and grew with a growth rate of 0.08 h^-1 ^(Figure 4). Thus, heterologous expression of *dld *from *C. glutamicum *enabled *C. efficiens *to utilize D-lactate as sole source of carbon and energy.

## Discussion

In this study *dld *(cg1027) was demonstrated to encode the only D-lactate dehydrogenase essential for the growth with D-lactate as sole carbon source in *C. glutamicum*. The *dld *inactivation mutant was unable to grow and to utilize D-lactate, unless *dld *was restored by plasmid-borne expression. The enzyme Dld is a quinone-dependent D-lactate dehydrogenase (EC 1.1.2.4). Dld is specific for D-lactate reduction, while D-malate, L-malate, D-tartrate and L-tartrate were not significant substrates. The determined *K_m _*of 0.62 mM for D-lactate is similar to D-lactate dehydrogenase from *Neisseria meningitidis *(0.7 mM [7]) and *E. coli *(0.49 mM [41]).

Dld accepts L-lactate and DL-2-hydroxybuytrate with minor activities confirming earlier observations obtained with strain DL4, a classically obtained mutant of *C. glutamicum *ATCC 14310 with increased D-lactate dehydrogenase activity and an increased rate of DL-hydroxybutyrate utilization [42]. Unpublished data on D-lactate dehydrogenase from strain DL4 (Scheer *et al. *as referred to in Bott & Niebisch [43]) revealed a pH optimum of 7.0, a Km for D-lactate of 0.15 mM and Vmax 0.26 U per mg of solubilized protein. This protein preparation contained non-covalently bound FAD as it was confirmed here for Dld from *C. glutamicum *ATCC 13032. As deduced from Dld of *E. coli *Dld of *C. glutamicum *also contains residues relevant for non-covalent FAD binding as well as those of the proposed active site (Ile-142 and Ser-144). Dld appears to be membrane-associated as Dld from *E. coli*, which does not contain transmembrane helices, but is firmly attached to the membrane by electrostatic interactions between an electropositive surface composed of several arginine and lysine residues in the membrane-binding domain and the electronegative phospholipid head groups of the membrane [44]. Dld from *C. glutamicum *contains several of these basic residues and was identified as a membrane associated protein in membrane proteome analyses [45]. Thus, it is tempting to speculate that membrane association of Dld could facilitate oxidation of D-lactate immediately after its uptake. As an uptake system for D- and/or L-lactate is currently unknown it cannot be tested whether Dld associates to the membrane and interacts with the uptake system.

Expression of *dld *is constitutive and independent of the carbon source as revealed by transcriptome analysis (Table 2) and specific D-lactate dehydrogenase activity measurements (Figure 2) confirming earlier observations [42]. Constitutive expression of *dld *as opposed to L-lactate inducible expression of the L-lactate dehydrogenase gene *lldD *[20] is also found in *E. coli *[46], while synthesis of L- and D-lactate dehydrogenases is regulated in a coordinated manner in *Acinetobacter calcoaceticus *[47].

**Table 2:** Comparative gene expression analysis of C. glutamicum ATCC 13032 grown in LB + D-lactate and LB or minimal media CgXII DL-lactate and CgXII L-lactate respectively.

**Gene**^**a**^	**Annotation**^**a**^	**mRNA level**^**b**^	
		LB	CgXII
cg0045	ABC-type transporter, permease component	**0,1**	n.d.
cg0594	ribosomal protein L3	1,3	**0,2**
cg0598	ribosomal protein L2	1,7	**0,2**
cg0652	ribosomal protein S13	0,9	**0,2**
cg0653	ribosomal protein S11	1,6	**0,2**
cg0769	ABC-type transporter, permease component	**0,2**	0,7
cg0771	ABC-type transporter, periplasmic component	0,3	0,7
cg0921	Siderophore-interacting protein	**0,2**	n.d.
cg1215	nicotinate-nucleotide pyrophosphorylase	1,0	**0,2**
cg1218	ADP-ribose pyrophosphatase	0,7	**0,2**
cg1351	molybdopterin biosynthesis enzyme	0,8	**0,2**
cg1362	F0F1-type ATP synthase a subunit	1,1	**0,2**
cg1366	F0F1-type ATP synthase alpha subunit	1,1	**0,2**
cg1447	Co/Zn/Cd efflux system component	**7,7**	0,7
cg1884	hypothetical protein	1,3	**0,2**
cg2402	cell wall-associated hydrolase	0,8	**0,2**
cg2931	putative dihydrodipicolinate synthase	**4,4**	1,0
cg2937	ABC-type transporter, periplasmic component	**4,6**	0,9
cg2938	ABC-type transporter, permease component	**4,1**	1,5
cg3114	sulfate adenylate transferase subunit 1	2,2	**0,2**
cg3116	phosphoadenosine phosphosulfate reductase	2,2	**0,1**
cg3118	putative nitrite reductase	2,3	**0,2**
cg3303	hypothetical protein	**4,0**	1,5

^a ^Gene identifiers and annotations are given according to BX927147.

^b ^Statistically significant changes of at least fourfold in gene expression determined in at least two independent experiments from independent cultivations (*P *< 0.05 by Student's test) are listed.

While *C. glutamicum *ATCC 13032 and ATCC 14310 contain D-lactate dehydrogenase Dld, the genome of *C. glutamicum *strain R does not encode Dld. Thus, *dld *is one of only 60 and 189 genes, respectively, that are strain-specific [48]. In addition, the gene *dld *is absent from the genomes of other corynebacterial species (*C. efficiens, C. jeikeium, C. urealytikum, C. diphtheriae, C. kroppenstedtii *and *C. aurimucosum*) as well as from the sequenced genomes of *Mycobacteriaceae *and of the sequenced genomes of other members of the suborder *Corynebacterineae *(*Dietziaceae, Gordoniaceae, Nocaridaceae *and *Tsukmurellaceae*). The genomic locus of *dld *(Figure 3) indicates that *dld *is flanked by the insertion elements IS*Cg6a *and IS*Cg6b *[49] and, thus, *dld *might have been acquired by horizontal gene transfer. The closest homolog of Dld from *C. glutamicum *is D-lactate dehydrogenase from *Propionibacterium freudenreichii *subsp. *shermanii*, which is encoded by PFREUD_16710 and shares 370 of 371 identical amino acids with Dld from *C. glutamicum*. Moreover, on the DNA level the genes and flanking sequences differ only by five nucleotides in 2372 bp region (bp 956767-959138 in GI 62388892/*C. glutamicum *and bp 1833090-1830719 in GI 297625198/*P. freudenreichii *subsp. *shermanii*). Insertion sequences with transposase genes belonging to the same family (family IS*3*) as those in the insertion sequences flanking *dld *in *C. glutamicum *can also be found adjacent to PFREUD_16710 in the genome of *P. freudenreichii *supporting the hypothesis of horizontal gene transfer between the two species. The G+C content of *dld *from *C. glutamicum *and PFREUD_16710 from *P. freudenreichii *is 62.2% and, thus, between the G+C content of the genomes of *C. glutamicum *(53.8%) and *P. freudenreichii *(67%; NC_014215). Meanwhile a horizontal transfer of *dld *from *E. coli *is likely excluded. The G+C-content of *dld *from *E. coli *is 51% which is close to G+C content of the *E. coli *genome (50%; NC_000913). Also the genomic context does not show any insertion sequences with transposase genes close to *dld*.

*P. freudenreichii *belongs to the suborder of *Propionibacterineae*, which along with other suborders such as the *Corynebacterineae *belongs to the order of *Actinomycetales*. Propionibacteria such as *P. freudenreichii *subsp. *shermanii *and corynebacteria such as *C. casei *are used in the dairy industry in cheese making and occur in the secondary flora of cheeses. In swiss-type cheese making, *P. freudenreichii *subsp. *shermanii *converts lactate anaerobically to propionate, acetate and carbon dioxide [1], while corynebacteria are involved in surface-ripening of red smear cheeses [50]. There is evidence for horizontal gene transfer between lactic acid bacteria fermenting milk (*Lactobacillus delbrueckii *subsp. *bulgaricus *and *Streptococcus thermophilus*; [51]. However, it is unclear under which conditions the horizontal transfer of *dld *between *C. glutamicum *and *P. freudenreichii *occurred although propionibacteria and corynebacteria are known to co-exist on the human skin [52].

Here we showed a functional heterologous expression of *dld *from *C. glutamicum *in *C. efficiens*. While the pBL1-based expression vector pEKEx3 [24] did not work in *C. efficiens *in our hands, the pHM1519-based expression vector pVWEx1 [34] may be used as a tool to extend the genetic repertoire of *C. efficiens *e.g. for a broader usage of different carbon sources.

The biotechnological production of lactic acid is observed with special interest due to its use for poly lactic acid production, an alternative to petroleum based plastic. Poly D-lactic acid (PDLA) is more advantageous than poly L-lactic acid (PLLA) because of its higher melting point [53]. While, poly lactic acid could be synthesized within recombinant *E. coli *cells [54], poly lactic acid is typically produced in a two step process. After fermentative production of lactic acid, poly lactic acid is synthesized chemically by ring-opening polymerisation of lactide, the cyclic diester of lactic acid [53]. Lactic acid fermentation employs lactic acid bacteria, but also *S. cerevisiae *has been engineered for production of high purity L-lactate [55] or D-lactate [56]. In addition, *E. coli *has been engineered for lactate production [57-59]. To improve D-lactate production by recombinant *E. coli*, *dld *was deleted to avoid re-utilization of the product [60]. As *C. glutamicum *strains other than ATCC 13032 lack *dld*, *C. glutamicum *might be a useful host for D-lactate production. Indeed, *C. glutamcium *R, which lacks *dld*, was engineered for D-lactate production under oxygen limiting conditions employing fermentative NAD-dependent D-lactate dehydrogenase from *E. coli *[28].

## Conclusion

Cg1067 encodes quinone-dependent D-lactate dehydrogenase Dld of *Corynebacterium glutamicum*. Dld is essential for growth with D-lactate as sole carbon source. The genomic region of *dld *likely has been acquired by horizontal gene transfer.

## Authors' contributions

OK and DM purified and characterized the enzyme, OK and KCS carried out the transcriptional studies, OK, KCS and JWY constructed the recombinant strains and JWY performed the growth experiments and determined the enzyme activities. TO supervised the enzymatic analyses, participated in the interpretation of the data and critical revision of the manuscript. VFW supervised the experiments and was responsible for the draft and final version of the manuscript. All authors read and approved the final manuscript.
